# Poly[aqua(μ-vinyl­phospho­nato)cadmium]

**DOI:** 10.1107/S160053681100780X

**Published:** 2011-03-15

**Authors:** Laura K. Byington Congiardo, Joel T. Mague, Aaron R. Funk, Ria Yngard, D. Andrew Knight

**Affiliations:** a Department of Chemistry, Florida Institute of Technology, Melbourne, FL 32901, USA; b Department of Chemistry, Tulane University, New Orleans, LA 70118, USA

## Abstract

The title compound, [Cd(C_2_H_3_O_3_P)(H_2_O)]_*n*_, was obtained from vinyl­phospho­nic acid and cadmium nitrate. The vinyl groups project into the inter­lamellar space and the structure is held together *via* van der Waals forces. The Cd^2+^ ion is six-coordinate and the geometry is best described as distorted octa­hedral, with O—Cd—O angles falling within the range 61.72 (13)–101.82 (14)°. Five of the coordinated oxygen atoms originate from the phospho­nate group and the sixth from a bound water molecule. Cd—O distances lie between 2.220 (3) and 2.394 (2) Å. The water mol­ecule is hydrogen bonded to a phospho­nate oxygen atom.

## Related literature

For the isotypic structure of [Zn(C_2_H_3_PO_3_)]·H_2_O, see: Menaa *et al.* (2002 ▶). For other cadmium organo­phospho­nates, see: Cao *et al.* (1993 ▶); Hou *et al.* (2008 ▶); Bauer *et al.* (2007 ▶). For other metal phospho­nates, see: Brody *et al.* (1984 ▶); Bujoli *et al.* (2001 ▶, 2007 ▶); Butcher *et al.* (2002 ▶); Cheetham *et al.* (1999 ▶); Clearfield *et al.* (1997 ▶); Clearfiled & Wang (2002 ▶); Fan *et al.* (2007 ▶); Hu *et al.* (2003 ▶). 
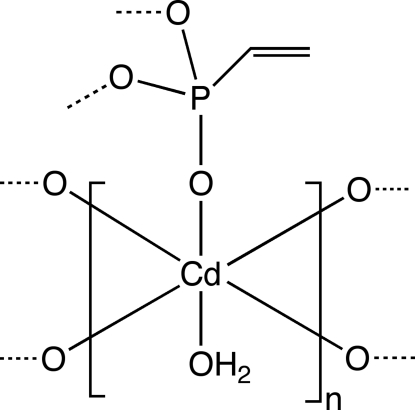


## Experimental

### 

#### Crystal data

[Cd(C_2_H_3_O_3_P)(H_2_O)]
*M* *_r_* = 236.43
Orthorhombic, 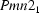

*a* = 5.9020 (7) Å
*b* = 9.7792 (12) Å
*c* = 4.9901 (6) Å
*V* = 288.01 (6) Å^3^
*Z* = 2
Mo *K*α radiation
μ = 3.99 mm^−1^
*T* = 100 K
0.12 × 0.11 × 0.01 mm

#### Data collection

Bruker APEX CCD area detector diffractometer
Absorption correction: multi-scan (*SADABS*; Sheldrick, 2008*a* ▶) *T*_min_ = 0.656, *T*_max_ = 0.956
2412 measured reflections
726 independent reflections
717 reflections with *I* > 2σ(*I*)
*R*_int_ = 0.021

#### Refinement

*R*[*F* ^2^ > 2σ(*F* ^2^)] = 0.020
*wR*(*F* ^2^) = 0.053
*S* = 1.16
726 reflections
46 parameters
7 restraints
H-atom parameters constrained
Δρ_max_ = 1.50 e Å^−3^
Δρ_min_ = −0.50 e Å^−3^
Absolute structure: Flack (1983 ▶), 303 Friedel pairs
Flack parameter: 0.05 (5)

### 

Data collection: *SMART* (Bruker, 2000 ▶); cell refinement: *SAINT-Plus* (Bruker, 2004 ▶); data reduction: *SAINT-Plus*; program(s) used to solve structure: *SHELXS97* (Sheldrick, 2008*b* ▶); program(s) used to refine structure: *SHELXL97* (Sheldrick, 2008*b* ▶); molecular graphics: *XP* in *SHELXTL* (Sheldrick, 2008*b* ▶) and *CrystalMaker* (CrystalMaker, 2010 ▶); software used to prepare material for publication: *publCIF* (Westrip, 2010 ▶).

## Supplementary Material

Crystal structure: contains datablocks I, global. DOI: 10.1107/S160053681100780X/pk2298sup1.cif

Structure factors: contains datablocks I. DOI: 10.1107/S160053681100780X/pk2298Isup2.hkl

Additional supplementary materials:  crystallographic information; 3D view; checkCIF report

## Figures and Tables

**Table 1:** Hydrogen-bond geometry (Å, °)

*D*—H⋯*A*	*D*—H	H⋯*A*	*D*⋯*A*	*D*—H⋯*A*
O1—H1*O*⋯O3^i^	0.84	2.12	2.916 (4)	158

Symmetry code: (i) 
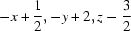
.

**Fig. 1.**
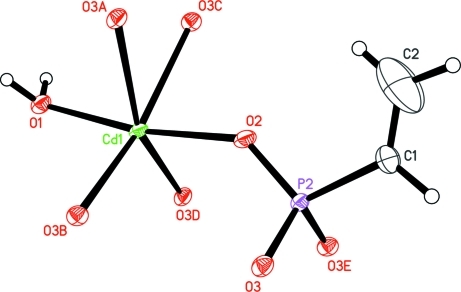
Thermal ellipsoid diagram (50% probability level) of [C2H3PO3Cd] . H2O. Symmetry-related P and O atoms with labels A - E are generated by the operations: A: x, y, 1-z; B: x, 2-y, z-0.5; C: -x, y, z-1; D: x-0.5, 2-y, z-0.5; E: -x, y, z

**Fig. 2.**
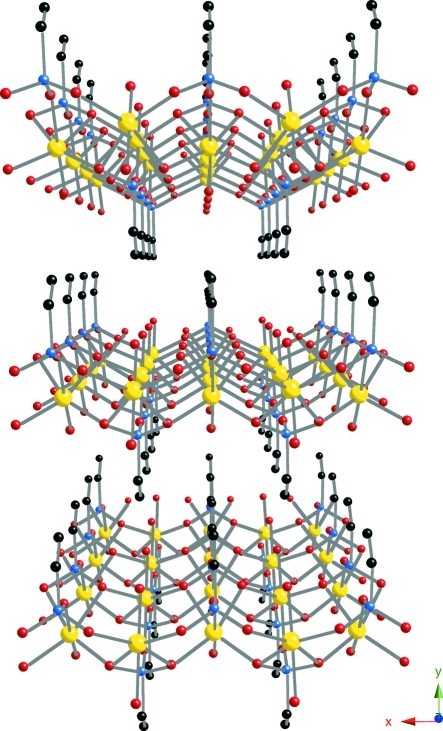
Representation of [C2H3PO3Cd] . H2O showing layered structure (view along c axis).

## supplementary crystallographic information

### Comment

Layered metal phosphonates as materials have potentially useful properties such as ion-exchange, catalysis, and homogeneous catalysis supports (Brody *et al.*, 1984;  Cheetham *et al.*, 1999; Clearfield *et al.*, 2002, 1997; Fan *et al.*, 2007). One of our recent objectives has been the preparation of covalently-bonded and catalytically active organometallic phosphonates which retain certain desirable features of a homogeneous catalyst (*e.g.* high activity and selectivity) and of the inorganic support (*e.g.* chemical and thermal stability, ease of catalyst grafting). These objectives may be realised *via* two possible methods: A. condensation of a pre-formed phosphonic acid functionalized coordination complex with di-, tri- or tetravalent metal salts, and B. post-synthetic modification of a layered metal phosphonate. Examples of Method A include TiO_2_-phosphonate supported rhodium bipyridine complexes for asymmetric hydrogenation of prochiral ketones (Bujoli *et al.*, 2001); ZrO_2_-phosphonate supported Ru-BINAP complexes for asymmetric hydrogenation of ketones and β-keto esters (Hu *et al.*, 2003), and TiO_2_-phosphonate supported cobalt phosphine carbonyl complexes for the hydroformylation of olefins (Bujoli *et al.*, 2007). In each of these examples, the catalytically active hybrid organometallic-inorganic phosphonate possesses quite different selectivities for organic transformation when compared to the homogeneous, unsupported counterparts. Examples of Method B are much rarer - no doubt in part due to the sterically constrained nature of layered metal phosphonates. The ability of such phosphonates to undergo a post-synthetic reaction with a catalytically active metal complex is dependent on the interlayer spacing present which is often only a few Ångstroms. Our own studies have shown that the interaction of metal vinylphosphonates C_2_H_3_PO_3_Cu and C_2_H_3_PO_3_Zn with rhodium(III) chloride in aqueous media does not result in the formation of an intact layered organometallic material but instead results in facile delamination of the layered phosphonate and which we tentatively ascribed to a Rh(III)-π-vinyl interaction (Butcher *et al.*, 2002). Herein we describe the synthesis, characterization and X-ray structure of a layered cadmium vinylphosphonate and subsequent reaction with rhodium chloride.

Single crystals of the title compound were obtained from the reaction of vinylphosphonic acid and cadmium nitrate tetrahydrate in water under conditions of slowly increasing pH. The structure is isotypic with that of the layered zinc analogue in which the vinyl groups project into the interlamellar space and is held together *via* Van der Waals forces (Menaa *et al.*, 2002). The Cd^2+^ ion is six-coordinate and the geometry is best described as distorted octahedral, with O—Cd—O angles falling within the range 61.72 (13) - 101.82 (14)°. Five of the coordinated oxygen atoms originate from the phosphonate group and the sixth, O3, from a bound water molecule. Cd—O distances lie between 2.220 (3) and 2.394 (2) Å, longer than those found in the zinc analog consistent with an increase in metal ionic radius, but similar to those found in previously reported layered cadmium organophosphonates. The structure of cadmium vinylphosphonate monohydrate is layered and Figure 2 shows a view down the *c* axis illustrating the lamellar nature of the material. The phosphorus atom is tetrahedrally coordinated, with a phosphorus-cadmium distance of 2.979 (1) Å. This longer than the Zn—P bond found in zinc vinylphosphonate (2.800 Å) which is predicted based on the larger cadmium ion (Menaa *et al.*, 2002). Two oxygen atoms from the same phosphonate –PO_3_ group chelate to the cadmium ion in a bidentate fashion. Each coordinated water molecule hydrogen bonds to oxygen atom O3 as listed in Table 2. The closest carbon-carbon interaction within a single layer is 4.990 (1) Å and across two layers is 3.816 (8) Å. The infra-red spectrum of [C_2_H_3_PO_3_Cd] ^.^ H_2_O recorded as a KBr pellet contains a single broad band centered at 3478 cm^-1^ corresponding to the cadmium-coordinated water O—H stretching mode and a band at 1614 cm^-1^ due to the bending mode. The spectrum also contains two intense bands at 1101 and 964 cm^-1^ correspond to –PO_3_ stretching modes and a weaker band at 747 cm^-1^ belonging to the monosubstituted vinyl moiety. These bands are similar to those found in hydrated copper vinylphosphonate (Butcher *et al.*, 2002). A thermal gravimetric analysis was also performed on crystals of [C_2_H_3_PO_3_Cd] ^.^ H_2_O and indicated weight losses of 8.0% at 189.1 °C and 7.1% at 520.2 °C which correspond to dehydration and loss of the vinyl portion of the phosphonate respectively. The reactivity of [C_2_H_3_PO_3_Cd] ^.^ H_2_O with rhodium(III) chloride was briefly investigated. A suspension of [C_2_H_3_PO_3_Cd] ^.^ H_2_O in aqueous rhodium chloride was allowed to react with stirring for several weeks under nitrogen. Disappearance of [C_2_H_3_PO_3_Cd] ^.^ H_2_O and formation of a rhodium mirror on the surface of the reaction flask suggested reduction of rhodium(III) to rhodium metal. The mechanism for this redox reaction is currently being investigated and will be reported in due course.

### Experimental

[Cd(C_2_H_3_PO_3_)] ^.^ H_2_O: A 1000 ml round bottom flask was charged with cadmium nitrate tetrahydrate (8.915 g, 28.90 mmol), vinylphosphonic acid (3.117 g, 28.85 mmol), and de-ionized water (175 ml). Urea (1.69 g, 28.2 mmol) was added to the solution, followed by an aqueous solution of NaOH (0.10 *M*), until the pH reached 2.8. The solution was heated in an oil-bath at 70 °C for 9 days. The resulting crystals were collected by filtration and dried in air to give [C_2_H_3_PO_3_Cd] ^.^ H_2_O as colorless plates (6.434 g, 94%). Anal. Calcd for C_2_H_5_CdO_4_P: C, 10.16; H, 2.13. Found: C, 10.43; H, 2.10.

### Refinement

H-atoms were placed in locations derived from a difference map and included as riding contributions with isotropic displacement parameters 1.2 times those of the attached atoms. Floating origin restraint required by space group. Atom C2 appears disordered across the mirror on the basis of its value for *U*_iso_ which is noticeably larger than that of C1. However, no satisfactory 2-site model could be devised to model this despite considerable effort. As a result, the displacement parameters for this atom was restrained to approximate isotropic behavior (ISOR 0.01).

### Crystal data

**Table:**

[Cd(C_2_H_3_O_3_P)(H_2_O)]	*F*(000) = 224
*M**_r_* = 236.43	*D*_x_ = 2.726 Mg m^−^^3^
Orthorhombic, *P**m**n*2_1_	Mo *K*α radiation, λ = 0.71073 Å
Hall symbol: P 2ac -2	Cell parameters from 2302 reflections
*a* = 5.9020 (7) Å	θ = 4.0–28.2°
*b* = 9.7792 (12) Å	µ = 3.99 mm^−^^1^
*c* = 4.9901 (6) Å	*T* = 100 K
*V* = 288.01 (6) Å^3^	Plate, colourless
*Z* = 2	0.12 × 0.11 × 0.01 mm

### Data collection

**Table:**

Bruker APEX CCD area detector diffractometer	726 independent reflections
Radiation source: fine-focus sealed tube	717 reflections with *I* > 2σ(*I*)
graphite	*R*_int_ = 0.021
φ and ω scans	θ_max_ = 28.0°, θ_min_ = 2.1°
Absorption correction: multi-scan (*SADABS*; Sheldrick, 2008*a*)	*h* = −7→7
*T*_min_ = 0.656, *T*_max_ = 0.956	*k* = −12→12
2412 measured reflections	*l* = −6→6

### Refinement

**Table:**

Refinement on *F*^2^	Secondary atom site location: difference Fourier map
Least-squares matrix: full	Hydrogen site location: inferred from neighbouring sites
*R*[*F*^2^ > 2σ(*F*^2^)] = 0.020	H-atom parameters constrained
*wR*(*F*^2^) = 0.053	*w* = 1/[σ^2^(*F*_o_^2^) + (0.0351*P*)^2^ + 0.0408*P*] where *P* = (*F*_o_^2^ + 2*F*_c_^2^)/3
*S* = 1.16	(Δ/σ)_max_ = 0.001
726 reflections	Δρ_max_ = 1.50 e Å^−^^3^
46 parameters	Δρ_min_ = −0.49 e Å^−^^3^
7 restraints	Absolute structure: Flack (1983), 303 Friedel pairs
Primary atom site location: heavy-atom method	Flack parameter: 0.05 (5)

### Special details

**Table:**

Geometry. All e.s.d.'s (except the e.s.d. in the dihedral angle between two l.s. planes) are estimated using the full covariance matrix. The cell e.s.d.'s are taken into account individually in the estimation of e.s.d.'s in distances, angles and torsion angles; correlations between e.s.d.'s in cell parameters are only used when they are defined by crystal symmetry. An approximate (isotropic) treatment of cell e.s.d.'s is used for estimating e.s.d.'s involving l.s. planes.
Refinement. Refinement of F2 against all reflections. The weighted *R*-factor *wR* and goodness of fit *S* are based on F2, conventional *R*-factors *R* are based on *F*, with *F* set to zero for negative F2. The threshold expression of F2 > 2 s(F2) is used only for calculating *R*-factors(gt) *etc*. and is not relevant to the choice of reflections for refinement. *R*-factors based on F2 are statistically about twice as large as those based on *F*, and *R*–factors based on all data will be even larger. Atom C2 appears disordered across the mirror on the basis of its value for *U*_iso_ which is noticeably larger than that of C1. However, no satisfactory 2-site model could be devised. CCDC-784849 contains the supplementary crystallographic data for this article. These data can be obtained free of charge at http://www.ccdc.cam.ac.uk/conts/retrieving.html [or from the Cambridge Crystallographic Data Center (CCDC), 12 Union Road, Cambridge CB2 1EZ, UK; fax: +44 (0)1223–336033; email: deposit@ccdc.cam.ac.uk].

### Fractional atomic coordinates and isotropic or equivalent isotropic displacement parameters (Å^2^)

**Table:**

	*x*	*y*	*z*	*U*_iso_*/*U*_eq_	
Cd1	0.0000	1.02972 (3)	0.10825 (10)	0.01148 (12)	
P2	0.0000	0.81874 (14)	0.6776 (2)	0.0107 (2)	
O1	0.0000	1.1926 (4)	−0.2266 (7)	0.0152 (7)	
H1O	0.1018	1.1927	−0.3443	0.018*	
O2	0.0000	0.8471 (4)	0.3805 (7)	0.0164 (8)	
O3	0.2081 (4)	0.8782 (3)	0.8230 (5)	0.0132 (5)	
C1	0.0000	0.6369 (6)	0.7329 (12)	0.0220 (12)	
H1	0.0000	0.6182	0.9433	0.026*	
C2	0.0000	0.5472 (8)	0.550 (3)	0.067 (4)	
H2A	0.0000	0.4540	0.6052	0.080*	
H2B	0.0000	0.5729	0.3668	0.080*	

### Atomic displacement parameters (Å^2^)

**Table:**

	*U*^11^	*U*^22^	*U*^33^	*U*^12^	*U*^13^	*U*^23^
Cd1	0.01300 (17)	0.01407 (18)	0.00737 (16)	0.000	0.000	−0.00103 (15)
P2	0.0131 (5)	0.0110 (5)	0.0080 (6)	0.000	0.000	0.0007 (4)
O1	0.0150 (18)	0.0198 (18)	0.0109 (17)	0.000	0.000	0.0009 (14)
O2	0.025 (2)	0.0153 (19)	0.0088 (19)	0.000	0.000	−0.0009 (15)
O3	0.0121 (12)	0.0167 (12)	0.0110 (10)	0.0003 (9)	0.0020 (10)	−0.0003 (8)
C1	0.030 (3)	0.013 (2)	0.023 (3)	0.000	0.000	0.001 (2)
C2	0.087 (7)	0.034 (4)	0.079 (8)	0.000	0.000	0.000 (4)

### Geometric parameters (Å, °)

**Table:**

Cd1—O3^i^	2.220 (3)	P2—Cd1^iv^	2.9791 (13)
Cd1—O2	2.244 (4)	O1—H1O	0.8400
Cd1—O1	2.309 (4)	O3—Cd1^v^	2.220 (3)
Cd1—O3^ii^	2.394 (2)	O3—Cd1^iv^	2.394 (2)
Cd1—P2^iii^	2.9791 (13)	C1—C2	1.265 (14)
P2—O2	1.508 (4)	C1—H1	1.0659
P2—O3	1.540 (3)	C2—H2A	0.9509
P2—C1	1.799 (6)	C2—H2B	0.9500
			
O3^i^—Cd1—O3^vi^	101.82 (14)	O2—P2—C1	109.4 (3)
O3^i^—Cd1—O2	91.76 (9)	O3—P2—C1	107.50 (16)
O3^i^—Cd1—O1	93.98 (9)	O2—P2—Cd1^iv^	125.58 (17)
O2—Cd1—O1	170.89 (13)	O3—P2—Cd1^iv^	53.05 (10)
O3^i^—Cd1—O3^ii^	159.44 (11)	C1—P2—Cd1^iv^	125.0 (2)
O3^vi^—Cd1—O3^ii^	98.05 (6)	Cd1—O1—H1O	120.4
O2—Cd1—O3^ii^	82.37 (10)	P2—O2—Cd1	137.8 (2)
O1—Cd1—O3^ii^	89.82 (11)	P2—O3—Cd1^v^	123.01 (15)
O3^vi^—Cd1—O3^iii^	159.44 (11)	P2—O3—Cd1^iv^	96.01 (12)
O3^ii^—Cd1—O3^iii^	61.72 (13)	Cd1^v^—O3—Cd1^iv^	115.72 (11)
O3^i^—Cd1—P2^iii^	128.99 (7)	C2—C1—P2	125.1 (7)
O2—Cd1—P2^iii^	83.42 (10)	C2—C1—H1	126.2
O1—Cd1—P2^iii^	87.47 (10)	P2—C1—H1	108.7
O3^ii^—Cd1—P2^iii^	30.94 (6)	C1—C2—H2A	117.2
O2—P2—O3	113.18 (14)	C1—C2—H2B	120.7
O3—P2—O3^vii^	105.8 (2)	H2A—C2—H2B	122.1
			
O3—P2—O2—Cd1	−60.15 (14)	C1—P2—O3—Cd1^v^	112.9 (2)
O3^vii^—P2—O2—Cd1	60.15 (14)	Cd1^iv^—P2—O3—Cd1^v^	−126.1 (2)
O3^i^—Cd1—O2—P2	50.94 (7)	O2—P2—O3—Cd1^iv^	118.03 (17)
O3^vi^—Cd1—O2—P2	−50.94 (7)	O3^vii^—P2—O3—Cd1^iv^	−6.4 (2)
O3^ii^—Cd1—O2—P2	−148.83 (7)	C1—P2—O3—Cd1^iv^	−121.04 (19)
O3^iii^—Cd1—O2—P2	148.83 (7)	O3—P2—C1—C2	−123.27 (12)
O2—P2—O3—Cd1^v^	−8.0 (3)	O3^vii^—P2—C1—C2	123.27 (12)
O3^vii^—P2—O3—Cd1^v^	−132.49 (11)		

Symmetry codes: (i) −*x*+1/2, −*y*+2, *z*−1/2; (ii) −*x*, *y*, *z*−1; (iii) *x*, *y*, *z*−1; (iv) *x*, *y*, *z*+1; (v) −*x*+1/2, −*y*+2, *z*+1/2; (vi) *x*−1/2, −*y*+2, *z*−1/2; (vii) −*x*, *y*, *z*.

### Hydrogen-bond geometry (Å, °)

**Table:**

*D*—H···*A*	*D*—H	H···*A*	*D*···*A*	*D*—H···*A*
O1—H1O···O3^viii^	0.84	2.12	2.916 (4)	158

Symmetry codes: (viii) −*x*+1/2, −*y*+2, *z*−3/2.
